# Hemodynamic Evaluation of Nonselective *β*-Blockers in Patients with Cirrhosis and Refractory Ascites

**DOI:** 10.1155/2018/4098210

**Published:** 2018-05-09

**Authors:** Alberto Ferrarese, Valerie Tikhonoff, Edoardo Casiglia, Paolo Angeli, Silvano Fasolato, Diego Faggian, Alberto Zanetto, Giacomo Germani, Francesco Paolo Russo, Patrizia Burra, Marco Senzolo

**Affiliations:** ^1^Multivisceral Transplant Unit, Department of Surgery, Oncology and Gastroenterology, Padua University Hospital, Padua, Italy; ^2^Department of Medicine, Padua University Hospital, Padua, Italy; ^3^Laboratory Medicine, Department of Medical and Surgical Sciences, University of Padua, Padua, Italy

## Abstract

**Background:**

Nonselective *β*-blockers (NSBB) have been associated with increased incidence of paracentesis-induced circulatory dysfunction (PICD) and reduced survival in patients with cirrhosis and refractory ascites.

**Aim:**

To prospectively evaluate a hemodynamic response to NSBB in cirrhotics listed for liver transplantation with refractory ascites undergoing large volume paracentesis (LVP).

**Methods:**

Patients with cirrhosis and refractory ascites, with an indication to start NSBB in primary prophylaxis for variceal bleeding, were enrolled. During two consecutive LVP, while being, respectively, off and on NSBB, cardiac output (CO), systemic vascular resistances (SVR), peripheral vascular resistances (PVR), and plasma renin activity (PRA) were noninvasively assessed.

**Results:**

Seventeen patients were enrolled, and 10 completed the study. Before NSBB introduction, SVR (1896 to 1348 dyn·s·cm^−5^; *p* = 0.028) and PVR (47 to 30 mmHg·min·dl·ml^−1^; *p* = 0.04) significantly decreased after LVP, while CO showed an increasing trend (3.9 to 4.5 l/m; *p* = 0.06). After NSBB introduction, LVP was not associated with a significant increase in CO (3.4 to 3.8 l/m; *p* = 0.13) nor with a significant decrease in SVR (2002 versus 1798 dyn·s·cm^−5^; *p* = 0.1). Incidence of PICD was not increased after NSBB introduction.

**Conclusion:**

The negative inotropic effect of NSBB was counterbalanced by a smaller decrease of vascular resistances after LVP, probably due to splanchnic *β*2-blockade. This pilot study showed that NSBB introduction may be void of detrimental hemodynamic effects after LVP in cirrhotics with refractory ascites.

## 1. Introduction

Ascites is the most frequent complication in the natural history of cirrhosis, and its development is significantly associated with impaired survival [1, 2]. Refractory ascites (RA) occurs in 5–10% of patients with cirrhosis and ascites, and it is associated with a significant worsening of central hemodynamics and a further reduction in survival [3]. Development of RA determines hyperdynamic circulation, peripheral vasodilation, and an impairment of cardiac and renal function [4].

In this clinical setting, several studies demonstrated that central hemodynamics could be further worsened by large volume paracentesis (LVP) in a wide proportion of patients (15% to 85%), leading to paracentesis-induced circulatory dysfunction (PICD), which has been associated with decreased survival and shortening of paracentesis-free interval time [5].

Nonselective beta blockers (NSBB) have been associated with a reduced survival in patients with RA [6], probably due to an increased incidence of PICD, suggesting a potential unfavorable mechanism linked with the worsening of central hemodynamics [7]. However, these data were in contrast with those reported by several studies, in which NSBB were associated with lower rate of liver decompensation [8], higher transplant-free survival [9], and a reduced risk of developing spontaneous bacterial peritonitis [10].

Thus, this prospective study aimed at assessing the role played by NSBB on central and peripheral hemodynamics in cirrhotics undergoing LVP for refractory ascites.

## 2. Materials and Methods

The study was conducted at the Padua University Hospital Liver Transplant (LT) Center, from December 2013 to December 2015.

All consecutive patients with cirrhosis listed for LT and diuretic-intractable or diuretic-resistant refractory ascites—according to the current guidelines [11]—undergoing repetitive LVP were evaluated. Exclusion criteria were noncirrhotic portal hypertension, ongoing chronic antihypertensive therapy, and nonadherence. Preliminary assessments of chronic obstructive pulmonary disease, asthma, severe bradycardia, and atrioventricular blocks were performed to rule out contraindication to NSBB use.

Each patient had to perform an oesophagogastroduodenoscopy within 6 months before enrollment. Those patients having a new indication to start NSBB for primary prophylaxis of variceal bleeding (e.g., patients with small varices with red wale marks or Child-Pugh C class; patients with medium-large varices) were enrolled. Propranolol was introduced at a starting dose of 40 mg twice daily and then adjusted according to hemodynamic parameters [12]. Repetitive LVP were consecutively performed by the same hepatologist and according to the current guidelines [11].

Central and peripheral hemodynamics were analyzed before and after two LVP, while being on and off NSBB therapy. Since hemodynamic measurement was not considered reliable in patients with bacterial infection or acute kidney injury, these patients were temporarily excluded from the study and eventually readmitted only after complete resolution of acute episodes.

Hemodynamic parameters, such as mean arterial pressure (MAP), cardiac output (CO), cardiac index (CI), heart rate (HR), were recorded through noninvasive techniques. MAP was measured (in mmHg) by a Finometer recorder (Finapres Medical Systems, Amsterdam, The Netherlands), a standalone solution for accurate automatic blood pressure system, monitoring and providing automatic heart rate recording. Cardiac function was measured through the amount of blood ejected from the left ventricle at each systole per minute (CO, l/min), by a cardiograph featuring enhanced bioimpedance signal morphology analysis, obtained through six leads positioned on the patient's thorax (PhysioFlow TM-Lab-1, Manatec Biomedical, Ebersviller, France) [13]. Arterial blood flow indexed for the volume of tissue was measured at a forearm with a strain gauge plethysmograph (Angioflow, Microlab Electronics, Padua, Italy). This method entails periodic occlusion of venous outflow by a cuff automatically inflated at overvenous and underdiastolic pressure, while the arm's volume is measured by indium-gallium-in silicone strain gauges. In such conditions, the segmental blood inflow is proportional to arterial flow, allowing real-time detection of peripheral flow [14–16]. Peripheral vascular resistances (PVR) were calculated (in mmHg × min × ml^−1^) from the mean blood pressure*/*forearm flow ratio, while systemic vascular resistances (in dyn × s × cm^−5^) were calculated from mean blood pressure*/*cardiac output ratio [14].

Plasma renin activity (PRA) was measured before LVP and one hour after the end of LVP from frozen plasma, using radioimmunoassay technique, similarly to previous studies [17]. Diagnosis of PICD was made after reaching an increase greater than 50% than pre-LVP values of PRA at 1 hour after the end of the procedure. This method was demonstrated to be as reliable as a measurement of PRA after 7 days of LVP for PICD detection [7].

All patients were followed up for 12 months, recording the outcome and eventual decompensation episodes.

All patients gave written informed consent at the time of enrollment. The study protocol was approved by the local ethical committee (n.2797P/2013). All diagnostic and therapeutic procedures belonging to the protocol were in accordance with the ethical guidelines of the Helsinki Declaration.

## 3. Statistical Analysis

Noncontinuous variables were assessed as frequencies and tested using Fisher's exact test, respectively. Continuous variables were assessed and tested as median (range) and compared using Mann–Whitney *U* test and Wilcoxon signed rank test to perform pairwise comparisons, respectively. A *p* value < 0.05 was considered statistically significant, whereas a trend towards significance was considered when *p* < 0.08. Analyses were performed with the SPSS statistical package (SPSS Inc. version 18.0, 2009, Chicago, IL, USA).

## 4. Results

### 4.1. Baseline Characteristics

**Figure 1:**
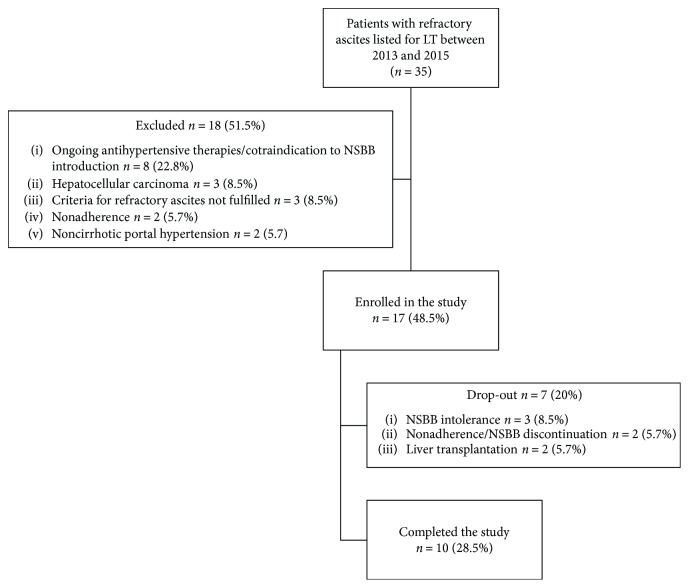
Patients' selection criteria and study design. NSBB: nonselective *β*-blockers. LT: liver transplantation.

During the study period, thirty-five patients with RA were prospectively evaluated, of whom 18 (51.4%) were not included for the following reasons: ongoing chronic antihypertensive therapies or contraindication to NSBB introduction (*n* = 8), hepatocellular carcinoma (*n* = 3), criteria for refractory ascites not fulfilled (*n* = 3), noncirrhotic portal hypertension (*n* = 2), and nonadherence (*n* = 2). A total of 17 patients (48.5%) were enrolled, and 10 completed the study. Causes of dropout were as follows: NSBB intolerance (*n* = 3), nonadherence/inappropriate discontinuation (*n* = 2), and liver transplantation (*n* = 2) (Figure 1).

All patients enrolled were listed for LT and had diuretic-intractable refractory ascites, which was treated with suboptimal diuretic therapy, due to the following: hyperkalemia (*n* = 4), impaired renal function (*n* = 4), and refractory encephalopathy (*n* = 2).

The median dose of NSBB was 60 mg/day (range 40–120). All patients fulfilled criteria for NSBB dose titration; NSBB dose was only temporarily reduced in one patient due to headache and hypotension, without complete withdrawal, and then titrated to achieve hemodynamic response. The median drainage volume was 7 liters per procedure (range 5–12). No significant intraindividual difference in the drained volume of ascites was found than previous procedure (7 [5–12] versus 7 [5–12], *p* = 1). During the study, spontaneous bacterial peritonitis was diagnosed in one patient; thus, hemodynamic parameters were not considered reliable and he was temporarily excluded from the study, until resolution of infection. The characteristics of patients enrolled in the study are summarized in Table 1.

### 4.2. Central and Peripheral Hemodynamics

**Figure 2:**
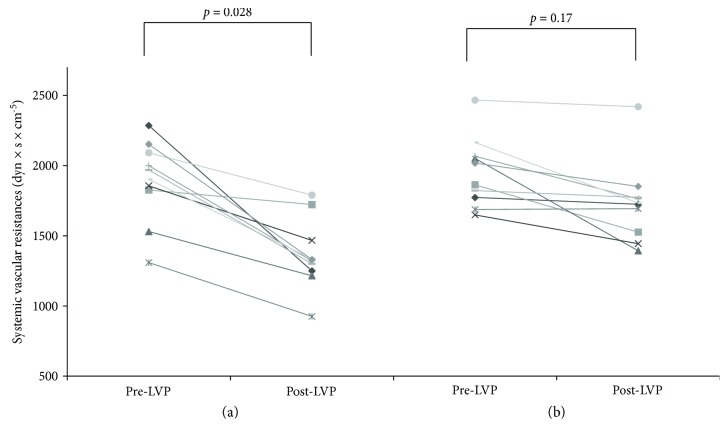
Systemic vascular resistances before and after large volume paracentesis: (a) before nonselective *β*-blocker introduction; (b) after nonselective *β*-blocker introduction.

**Figure 3:**
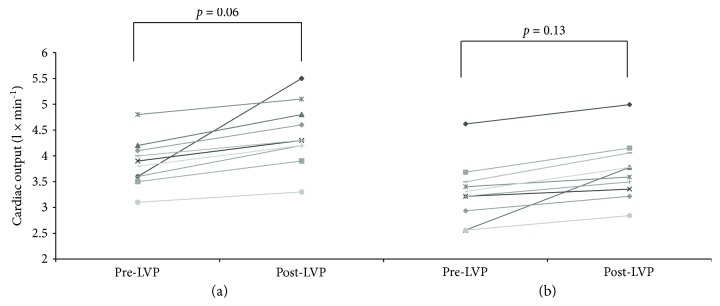
Cardiac output before and after large volume paracentesis: (a) before nonselective *β*-blocker introduction; (b) after nonselective *β*-blocker introduction.

Before NSBB introduction, SVR showed a significant reduction after LVP (1896 [1276–2293] versus 1348 [925–1804] dyn × s × cm^−5^; *p* = 0.028; Figure 2(a)). Similarly, MAP significantly decreased (82 [71–103] versus 72 [68–86] mmHg, *p* = 0.03), whereas CO showed an increasing trend (3.9 [2.8–4.8] versus 4.5 [3.1–4.9] l/m; *p* = 0.06; Figure 3(a)). Furthermore, there was a reduction of PVR (47 [36–54] versus 30 [22–33] mmHg × min × dl × ml^−1^; *p* = 0.04). PICD was diagnosed in 2/10 (20%) patients, after an increase in PRA more than 50% of the pre-LVP values. There was no significant difference between PRA values before and after LVP (9.4 [5.5–14.2] versus 13 [6.4–16.9] ng × ml^−1^ × h^−1^; *p* = 0.09).

Patients underwent the second LVP after a mean time of 10 (9–12 days) from NSBB introduction. At baseline, no significant differences were found on hemodynamics than before previous LVP, except for heart rate, which was significantly decreased (76 [range 68–94] versus 63 [range 55–73] bpm; *p* = 0.05) after NSBB introduction (Table 2).

When performing LVP on NSBB therapy, there was no significant reduction of SVR (2002 [range 1609–2542] versus 1798 [range 1382–2863] dyn × s × cm^−5^; *p* = 0.17; Figure 2(b)) nor significant increase of CO (3.4 [range 2.2–4.8] versus 3.8 [range 2.1–5.1] l/m; *p* = 0.13, Figure 3(b)) and of PVR (49 [range 28–53] versus 36 [22–44] mmHg × min × dl × ml^−1^; *p* = 0.2). Nevertheless, MAP significantly decreased after LVP (83 [range 60–96] versus 78 [range 58–85] mmHg; *p* = 0.05).

PRA increased 50% more than pre-LVP values in 3 patients, without a significant increase in the whole cohort (6.3 [4.8–9.8] versus 8.6 [4.9–11.6] ng × ml^−1^ × h^−1^; *p* = 0.07). No significant incidence of PICD was found in comparison with the previous LVP.

Patients were followed up for 12 months. All patients continued NSBB and underwent LVP for RA. During the follow-up, three patients developed spontaneous bacterial peritonitis (median time since NSBB introduction: 110 days [range 10–155]). Hepatorenal syndrome occurred in two patients after two episodes of bacterial infection (pneumonia and spontaneous bacterial peritonitis, resp.) at a median time of 144 days from enrollment. No episode of variceal bleeding occurred. Cumulative mortality at one year was 40% (3 patients died of liver failure, 1 patient of cerebral hemorrhage). Two patients underwent liver transplantation, and one patient dropped out the waiting list due to alcohol relapse.

## 5. Discussion

LVP in patients with cirrhosis and RA further enhances the preexisting hyperdynamic circulation, via a counter-regulatory overactivation of vasoconstrictor systems. This can cause renal hypoperfusion and finally PICD [18, 19].

Data we presented were consistent with those previously reported on patients undergoing LVP, in whom the reduction of systemic and peripheral vascular resistances has been clearly demonstrated [20].

In the setting of RA, it has been postulated that NSBB could produce further derangements on hemodynamics and decrease cardiac chronotropic competence [21]. In our study, NSBB introduction did not impair central hemodynamics after LVP. The lower increase on CO (3.4 to 3.8 l/m) was associated with a reduced postparacentesis splanchnic vasodilation. The *β*2-blockade might determine a splanchnic vasoconstriction with a smaller post-LVP decrease of SVR; thus, a smaller increase of CO should be required to counterbalance hemodynamic changes induced by LVP.

Regarding hemodynamics, our data were different from those highlighted in the study by Sersté et al. [7], (in which 10/11 patients developed PICD), but in line with other studies [22–24]. Furthermore, SVR and CO were noninvasively assessed by bioimpedance analysis, whose reproducibility and accuracy have been already demonstrated [13, 14, 25, 26]. A study in 45 patients with cirrhosis (half of them with ascites) demonstrated that total-body bioimpedance analysis provided reliable and reproducible data regarding compartmental volume distribution [27]. Even though this method has not been validated yet for the assessment of CO in patients with RA, data provided in our study were similar to what already demonstrated with invasive techniques [20]. Moreover, data were collected in the same patient to avoid interindividual variability and were obtained just from a thoracic bioimpedance analysis, not being influenced by ascites and/or lower limb edema, as in other total-body techniques. Lastly, a noninvasive measurement of central and peripheral hemodynamics was required due to the design of the study, by which 4 invasive hemodynamic measurements would be performed in the same patient.

We can hypothesize that NSBB could produce a new, although not enough stable, equilibrium, through which systemic organ perfusion is not going impaired unless when undergoing elevated stressing events. Recently, several studies provided data on the role of NSBB in decompensated cirrhosis [22, 28]; Krag et al. [29] resumed these findings hypothesizing a window therapy, which was also discussed and modified afterwards [30]. This hypothesis, which seems to reduce the use of NSBB in cirrhosis, “cutting” the sickest decompensated patients, remains still matter of discussion amongst hepatologists [31]. During the follow-up, two patients developed two episodes of HRS after bacterial infection. Although a clinical interpretation could not be drawn due to the small cohort, this observation is in concordance with the data by Mandorfer et al. [9], who showed a 20% cumulative incidence of HRS after SBP development in cirrhotics treated with NSBB.

The main limitations of this study were the small sample size, mainly due to strict clinical criteria. The fact that only 58% of patients completed the study because nonadherence or NSBB intolerance is commonly seen in clinical practice in patients with decompensated cirrhosis [32]. However, all patients who completed the study showed a reduction of heart rate greater than 25% after NSBB introduction.

In conclusion, the negative inotropic effect of NSBB seems to be counterbalanced by a smaller decrease of vascular resistances after LVP, probably due to splanchnic *β*2-blockade. This pilot study showed that NSBB introduction may be void of detrimental hemodynamic effects after LVP in cirrhotics with refractory ascites.

## Figures and Tables

**Table 1:** Characteristics of enrolled patients. Continuous variables are expressed as median (range). BMI: body mass index.

Gender (males), *n* (%)	4 (40)
Age (years)	57.5 (45–68)
Etiology of liver disease, *n* (%)	
Alcoholic	3 (30)
HCV	2 (20)
HBV	1 (10)
Mixed	4 (40)
BMI	22.7 (19–33)
Serum bilirubin (mg/dl)	1.2 (0.6–3.5)
Serum creatinine (mg/dl)	1.2 (0.6–1.4)
Serum albumin (mg/dl)	34 (33–40)
Previous overt hepatic encephalopathy, *n* (%)	8 (80%)
MELD score	11 (9–23)
MELD-Na score	16 (14–26)
Child-Pugh score classes, *n* (%)	
A	0
B	4 (40)
C	6 (60)
Propranolol titration dose (mg/day)	60 (40–120)
Diuretic therapy (mg/day)	
Potassium canrenoate	200 (100–300)
Furosemide	50 (50–100)
Liters of ascites removed per procedure	7 (5–12)

**Table 2:** Intraindividual changes of hemodynamic parameters before and after LVP, while being on and off NSBB. Values are expressed as median (range). HR: heart rate; MAP: mean arterial pressure; CO: cardiac output; CI: cardiac index; SVR: systemic vascular resistance; PVR: peripheral vascular resistance; PRA: plasma renin activity. ^a^Pre-LVP versus pre-LVP with and without NSBB. ^b^Post-LVP versus post-LVP with and without NSBB.

	Absence of NSBB			Presence of NSBB			*p* value^a^	*p* value^b^
	Pre-LVP	Post-LVP	*p* value	Pre-LVP	Post-LVP	*p* value	—	
HR (bpm)	76 [68–94]	83 [76–89]	0.13	63 [55–73]	74 [60–85]	**0.02**	**0.05**	0.08
MAP (mmHg)	82 [71–103]	72 [68–86]	**0.03**	83 [60–96]	78 [58–85]	**0.05**	0.3	0.3
CO (l/min)	3.9 [2.8–4.8]	4.5 [3.7–4.9]	0.06	3.4 [3–3.9]	3.8 [3.5–4.1]	0.13	0.2	0.09
CI (l/min/m^2^)	2.4 [2.1–2.9]	2.8 [2.1–3.1]	0.07	2.1 [2–2.5]	2.3 [2.1–2.9]	0.34	0.5	0.09
SVR (dyn·s·cm^−5^)	1896 [1276–2293]	1348 [925–1804]	**0.028**	2002 [1609–2542]	1798 [1382–2863]	0.17	0.5	0.07
PVR (mmHg·min·dl·ml^−1^)	47 [36–54]	30 [22–30]	**0.04**	49 [27–53]	36 [21–44]	0.2	0.8	0.09
PRA (ng·ml^−1^·h^−1^)	9.4 [5.3–14.2]	13 [6.4–16.9]	0.09	6.3 [4.8–9.6]	8.6 [4.9–11.6]	0.07	0.07	0.07

## Data Availability

The data used to support the findings of this study are available from the corresponding author upon request.

## Abbreviations

CO: Cardiac output
CI: Cardiac index
HR: Heart rate
LVP: Large volume paracentesis
MAP: Mean arterial pressure
NSBB: Nonselective beta blockers
PRA: Plasma renin activity
PICD: Paracentesis-induced circulatory dysfunction
PVR: Peripheral vascular resistances
RA: Refractory ascites
SBP: Spontaneous bacterial peritonitis
SVR: Systemic vascular resistances.

## References

[1] Runyon B. A. (2009). Management of adult patients with ascites due to cirrhosis: an update.

[2] D'Amico G., Morabito A., D'Amico M. (2018). Clinical states of cirrhosis and competing risks.

[3] Moreau R., Delegue P., Pessione F. (2004). Clinical characteristics and outcome of patients with cirrhosis and refractory ascites.

[4] Bosch J., Groszmann R. J., Shah V. H. (2015). Evolution in the understanding of the pathophysiological basis of portal hypertension: how changes in paradigm are leading to successful new treatments.

[5] Ginès A., Fernández-Esparrach G., Monescillo A. (1996). Randomized trial comparing albumin, dextran 70, and polygeline in cirrhotic patients with ascites treated by paracentesis.

[6] Sersté T., Melot C., Francoz C. (2010). Deleterious effects of beta-blockers on survival in patients with cirrhosis and refractory ascites.

[7] Sersté T., Francoz C., Durand F. (2011). Beta-blockers cause paracentesis-induced circulatory dysfunction in patients with cirrhosis and refractory ascites: a cross-over study.

[8] Hernández-Gea V., Aracil C., Colomo A. (2012). Development of ascites in compensated cirrhosis with severe portal hypertension treated with *β*-blockers.

[9] Mandorfer M., Bota S., Schwabl P. (2014). Nonselective *β* blockers increase risk for hepatorenal syndrome and death in patients with cirrhosis and spontaneous bacterial peritonitis.

[10] Senzolo M., Cholongitas E., Burra P. (2009). *β*-Blockers protect against spontaneous bacterial peritonitis in cirrhotic patients: a meta-analysis.

[11] Moore K. P., Wong F., Gines P. (2003). The management of ascites in cirrhosis: report on the consensus conference of the International Ascites Club.

[12] Garcia-Pagan J. C., Grace N. D. (2001). Session 5 – primary prophylaxis.

[13] Richard R., Lonsdorfer-Wolf E., Charloux A. (2001). Non-invasive cardiac output evaluation during a maximal progressive exercise test, using a new impedance cardiograph device.

[14] Casiglia E., Palatini P., Colangeli G. (1996). 24 h rhythm of blood pressure and forearm peripheral resistance in normotensive and hypertensive subjects confined to bed.

[15] Casiglia E., Ginocchio G., Tikhonoff V. (2000). Blood pressure and metabolic profile after surgical menopause: comparison with fertile and naturally-menopausal women.

[16] Casiglia E., Pizziol A., Tikhonoff V. (2000). The 24-hour rhythm of blood pressure differs from that of leg hemodynamics in orthotopic heart transplant recipients.

[17] Ginès P., Arroyo V., Vargas V. (1991). Paracentesis with intravenous infusion of albumin as compared with peritoneovenous shunting in cirrhosis with refractory ascites.

[18] Coll S., Vila M. C., Molina L., Gimenez M. D., Guarner C., Solà R. (2004). Mechanisms of early decrease in systemic vascular resistance after total paracentesis: influence of flow rate of ascites extraction.

[19] Sola-Vera J., Miñana J., Ricart E. (2003). Randomized trial comparing albumin and saline in the prevention of paracentesis-induced circulatory dysfunction in cirrhotic patients with ascites.

[20] Vila M. C., Solà R., Molina L. (1998). Hemodynamic changes in patients developing effective hypovolemia after total paracentesis.

[21] Krag A., Møller S., Burroughs A. K., Bendtsen F. (2012). Betablockers induce cardiac chronotropic incompetence.

[22] Reiberger T., Mandorfer M. (2017). Beta adrenergic blockade and decompensated cirrhosis.

[23] Nasr G., Hassan A., Ahmed S., Serwah A. (2010). Predictors of large volume paracantesis induced circulatory dysfunction in patients with massive hepatic ascites.

[24] Appenrodt B., Wolf A., Grünhage F. (2008). Prevention of paracentesis-induced circulatory dysfunction: midodrine vs albumin. A randomized pilot study.

[25] Kalantari K., Chang J. N., Ronco C., Rosner M. H. (2013). Assessment of intravascular volume status and volume responsiveness in critically ill patients.

[26] Piccoli A., for the Italian Hemodialysis-Bioelectrical Impedance Analysis (HD-BIA) Study Group (1998). Identification of operational clues to dry weight prescription in hemodialysis using bioimpedance vector analysis.

[27] Davenport A., Argawal B., Wright G. (2013). Can non-invasive measurements aid clinical assessment of volume in patients with cirrhosis?.

[28] Mookerjee R. P., Pavesi M., Thomsen K. L. (2016). Treatment with non-selective beta blockers is associated with reduced severity of systemic inflammation and improved survival of patients with acute-on-chronic liver failure.

[29] Krag A., Wiest R., Albillos A., Gluud L. L. (2012). The window hypothesis: haemodynamic and non-haemodynamic effects of *β*-blockers improve survival of patients with cirrhosis during a window in the disease.

[30] Ge P. S., Runyon B. A. (2014). The changing role of beta-blocker therapy in patients with cirrhosis.

[31] Ferrarese A., Tsochatzis E., Burroughs A. K., Senzolo M. (2014). Beta-blockers in cirrhosis: therapeutic window or an aspirin for all?.

[32] Thalheimer U., Christie J., Burroughs A. K. (2012). Benefits of beta blockade beyond bleeding prophylaxis and the role of adherence.

